# Volumetric Temperature Mapping Using Light-Sheet Microscopy and Upconversion Fluorescence from Micro- and Nano-Rare Earth Composites

**DOI:** 10.3390/mi14112097

**Published:** 2023-11-14

**Authors:** Dannareli Barron-Ortiz, Ruben D. Cadena-Nava, Enric Pérez-Parets, Jacob Licea-Rodriguez, Emilio J. Gualda, Juan Hernandez-Cordero, Pablo Loza-Alvarez, Israel Rocha-Mendoza

**Affiliations:** 1 Centro de Investigación Científica y de Educación Superior de Ensenada (CICESE), Carretera Ensenada-Tijuana, No. 3918, Zona Playitas, Ensenada 22860, Mexico; dbarron@cicese.edu.mx; 2 Centro de Nanociencias y Nanotecnología (CNyN), Universidad Nacional Autónoma de México (UNAM), Km 107 Carretera Tijuana-Ensenada, Pedregal Playitas, Ensenada 22860, Mexico; rcadena@ens.cnyn.unam.mx; 3 ICFO-Institut de Ciencies Fotoniques, The Barcelona Institute of Science and Technology, Av. Carl Friedrich Gauss, 3, 08860 Castelldefels, Spain; 4 Centro de Investigación en Ingeniería y Ciencias Aplicadas (CIICAp), Universidad Autónoma del Estado de Morelos, Cuernavaca 62209, Mexico; 5 Department of Agri-Food Engineering and Biotechnology (DEAB), Universitat Politècnica de Catalunya, Esteve Terradas 8, 08860 Castelldefels, Spain; emilio.jose.gualda@upc.edu; 6 Instituto de Investigaciones en Materiales, Universidad Nacional Autónoma de México, A.P. 70-360, Mexico City 04510, Mexico; jhcordero@iim.unam.mx

**Keywords:** light-sheet microscopy, upconversion fluorescence, 3D imaging, temperature mapping

## Abstract

We present a combination of light-sheet excitation and two-dimensional fluorescence intensity ratio (FIR) measurements as a simple and promising technique for three-dimensional temperature mapping. The feasibility of this approach is demonstrated with samples fabricated with sodium yttrium fluoride nanoparticles co-doped with rare-earth ytterbium and erbium ions (NaYF_4_:Yb^3+^/Er^3+^) incorporated into polydimethylsiloxane (PDMS) as a host material. In addition, we also evaluate the technique using lipid-coated NaYF_4_:Yb^3+^/Er^3+^ nanoparticles immersed in agar. The composite materials show upconverted (UC) fluorescence bands when excited by a 980 nm near-infrared laser light-sheet. Using a single CMOS camera and a pair of interferometric optical filters to specifically image the two thermally-coupled bands (at 525 and 550 nm), the two-dimensional FIR and, hence, the temperature map can be readily obtained. The proposed method can take optically sectioned (confocal-like) images with good optical resolution over relatively large samples (up to the millimetric scale) for further 3D temperature reconstruction.

## 1. Introduction

Whether in life science scientific research, medical diagnostics, or the characterization of microelectromechanical devices, taking reliable and accurate remote temperature measurements of specific areas or volumes is of great interest. For instance, in microbiology, mapping different organelle temperatures can be useful for gaining a better understanding of intracellular events since biological cells in the body constantly generate energy and thermal effects [1,2]; in medicine, mapping temperature deviations from the basal temperature of tissues and organs while a metabolic activity, an acute trauma, an infection-induced inflammation, or any other pathological condition occurs could be useful in gaining a better understanding of the onset and development of these processes [3,4]; meanwhile, in the case of microdevice characterization during the fabrication process, surface temperature homogeneity could be assessed [5,6].

Since fluorescence emission is a temperature-dependent process, several optical methods for remote temperature sensing exist nowadays based on the temperature dependence of different specific fluorescence parameters [7], among them, the lifetime [1,3,8,9], the spectral band shift [2], and the intensity value of a specific band [4,10]. Temperature imaging in biological systems has been successfully demonstrated using imaging methods in 2D [1,8] or even 3D [2]. However, these methods require precise time synchronization for fluorescent lifetime detection or point spread function engineering combined with a spectral shift analysis, which makes these imaging systems relatively complex to implement.

Another fluorescence parameter used for remote temperature sensing, on which we based this work, is the intensity ratio measured from a pair of thermally coupled fluorescence spectral bands known as the fluorescence (or luminescence) intensity ratio, called FIR (or LIR) for short. FIR measurements have been widely used in recent years as a technique for optical thermometry given their inherent features [11,12,13,14,15,16,17], such as noise cancellation, real-time temperature sensing, and high sensitivity, making them very attractive for performing remote 2D optical thermometry since, in principle, such bidimensional temperature measurements are possible by taking specific images of the two thermally coupled fluorescent bands. For instance, Sedmark et al. [5] performed microscopic imaging of local temperature distributions within a transparent rare-earth-doped glass–ceramic; Van Swieten et al. [6] accurately mapped the temperature profile of a MEMS-based microheater using chemically stable upconverted nanoparticles (UC NPs) composed of sodium yttrium fluoride (NaYF_4_) co-doped with rare-earth elements; and Vetrone et al. [18] measured temperature gradients in water-dispersible PEI-capped NaYF_4_:Er^3+^/Yb^3+^ NP solutions. The images in these works were obtained using confocal microscopy by raster scanning the focused excitation beam while performing point-to-point detection, which can be time-consuming depending on the signal efficiency and scan speed. Such confocal acquisition allowed for three-dimensional imaging capabilities but in small volumes [5,6].

An alternative imaging technique for rapid 3D imaging is light-sheet fluorescent microscopy (LSFM) [19,20,21,22,23,24,25,26,27]. In LSFM, a light plane selectively excites the luminescent material at a specific sample plane, from which the induced fluorescence is orthogonally collected via a fast CMOS camera [19,20,28,29,30]. Such a configuration permits the acquisition of high-speed optically sectioned images with good lateral and axial resolution for rapid 3D visualization. In contrast to confocal laser scanning microscopy (CLSM), LSFM has proven to be a powerful tool for in vivo time-lapse studies of relatively large biological samples such as the fish Medaka [19] and zebrafish [22,25], *Drosophila* melanogaster embryos [27], mouse brains [21], *Caenorhabditis elegans* [23,24], and tumor cell spheroids [26], among many others.

In this context, this work demonstrates that performing FIR measurements on temperature-dependent upconverting fluorescent composites using LSFM is a simple and cost-effective way, compared with CLSM, to perform 2D and 3D temperature imaging in either short or relatively large regions, ranging from tens of microns up to tens of millimeters, and to preserve good enough optical resolution. We show two- and three-dimensional temperature mapping distributions on temperature sensors [12] (acting as a large sample) composed of polydimethylsiloxane and sodium yttrium fluoride co-doped with rare-earth ytterbium and erbium elements (PDMSd-NaYF_4_:Yb^3+^/Er^3+^), and samples containing lipid-coated NaYF_4_:Yb^3+^/Er^3+^ nanoparticles immersed in agar (as short samples). Our results show that light-sheet imaging has excellent potential to perform 3D microscopy thermometry over large biological systems that can allocate functionalized UC NPs.

## 2. Theoretical Background

### 2.1. Upconversion from NaYF_4_:Yb^3+^/Er^3+^

**Figure 1:**
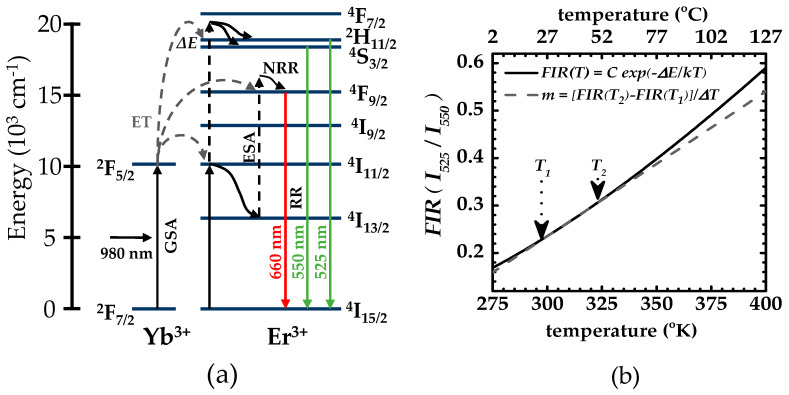
(**a**) Energy level diagram and upconversion process of a sensitizer/activator-coupled system for NaYF_4_:Yb^3+^/Er^3+^ materials. In the figure, the ground state absorption (GSA) is represented by black solid arrows, the excited state absorption (ESA) by black dashed arrows, the energy transfer (ET) by gray dashed arrows, the nonradiative relaxation (NRR) by black wavy arrows, and radiative relaxation (RR) by red and green solid arrows. (**b**) FIR temperature dependence. The solid line shows the characteristic exponential growth of NaYF_4_:Yb^3+^/Er^3+^ materials, and the dashed line shows the linear fit approximation around the 25 to 50 °C temperature range.

Upconversion luminescence is a process where two or more low-energy (long-wavelength) photons are sequentially absorbed, leading to higher-energy (short-wavelength) photon emission. For upconversion generation in the visible region through infrared light (IR) excitation, inorganic host crystals such as hexagonal sodium yttrium fluoride (NaYF_4_) doped with single erbium (Er^3+^) ions or co-doped with erbium (Er^3+^) and ytterbium (Yb^3+^) (for IR cross-section absorption enhancement) are the most efficient materials [15,31,32]. The energy levels of such a UC system, depicted in Figure 1, lead to the emission of three characteristic fluorescence bands centered at around 525, 550, and 660 nm when the composite material is irradiated at 980 nm [16,31,32,33]. Given the low water absorption of 980 nm near-infrared excitation light (with a coefficient absorption of around 0.4 cm^−1^ [34]) and given the good quantum efficiency (typically > 59%) and fast response time of CMOS cameras for green/red signal detection, along with the excellent transparency of visible light in water, NaYF_4_:Yb^3+^/Er^3+^ materials are potential candidates for imaging various thermal processes in biomedical science applications [12,18,31,35].

Figure 1a shows a typical energy level diagram and the upconversion process of a sensitizer/activator-coupled system for NaYF_4_:Yb^3+^/Er^3+^ material. The main mechanisms involved in the process are ground state absorption (GSA), excited state absorption (ESA), energy transfer (ET), nonradiative relaxation (NRR), and radiative relaxation (RR). In NaYF_4_:Yb^3+^/Er^3+^ materials, 20% of the Na/Y^3+^ ions are usually replaced with Yb^3+^ and 3% by Er^3+^ in the original NaYF_4_ hexagonal lattice; therefore, Yb^3+^ acts as the main IR light absorber (sensitizer) and Er^3+^ as the visible light emitter (activator) in the upconversion process. Notice that the ET and ESA mechanisms are based on the sequential absorption of two or more photons by the metastable (long-lived) energy states of the Er ions conferred by the crystal lattice of the host NaYF_4_ [31]. First, Yb^3+^ absorbs 980 nm photon energy via GSA, promoting the ion transition ^2^F_7/2_ **→** ^2^F_5/2_. Then, an ET from Yb^3+^ to Er^3+^ enables ^4^I_11/2_ state excitation. Alternatively, the GSA of Er^3+^ can also occur, promoting the ion transition ^4^I_15/2_ → ^4^I_11/2_. Once in the ^4^I_11/2_ state, the Er^3+^ can go through to either an NRR or another ET mechanism. In the former, the ion relaxes non-radiatively down to the ^4^I_13/2_ state followed by a second ET from Yb^3+^ to Er^3+^ [16,31,32,33,36], promoting the ion to the ^4^F_9/2_ state (via ESA and NRR mechanisms) and then relaxes down to the Er^3+^ ground state via an RR mechanism enabling the ^4^F_9/2_ **→** ^4^I_15/2_ transition and emitting the 660 nm photon (note that the mechanism for red upconversion is not universally agreed upon and can be described as a two-photon [15,36,37] or three-photon process [15,38]). In the latter, the other ET promotes the ion to the ^2^H_11/2_ and ^4^S_3/2_ states (again via ESA and NRR mechanisms), subsequently relaxing down radiatively to the Er^3+^ ground state, promoting the ^4^S_4/2_ **→** ^4^I_15/2_ transition for the 550 nm photon emission and the ^2^H_11/2_ **→** ^4^I_15/2_ transition for the 525 nm photon emission.

### 2.2. Fluorescence Intensity Ratio in NaYF_4_:Yb^3+^/Er^3+^

When two energy levels are separated by less than 2000 cm^−1^ (<0.248 eV), they are said to be thermally coupled since the higher energy level can be occupied at the expense of the lower level by a simple increase in thermal energy [17]. In the NaYF_4_:Yb^3+^/Er^3+^ system, the energy band gap, ∆E, between the ^2^H_11/2_ and ^4^S_4/2_ levels of Er^3+^ is approximately 866 cm^−1^ ($=(1/525-1/550)\times 10^{-7}{cm}^{-1}$), so these energy levels are thermally coupled. The ratio between their integrated intensities follows a Boltzmann-type population distribution; therefore, their fluorescence intensity ratio is provided by

(1) $$FIR(T)=I_{525}/I_{550}=Cexp\left(\frac{-∆E}{kT}\right)$$

where *I*_550_ and *I*_525_ are the integrated intensities of each band; ∆E is the energy difference between the ^2^H_11/2_ and ^4^S_4/2_ levels; k is the Boltzmann constant (0.695 cm^−1^/°K); T is the absolute temperature in Kelvin (°K); and C is a constant associated with the host materials and formed by the degeneracy of the coupled energy levels, their emission frequencies, and their spontaneous radiation transition rates [14,17,39,40,41] Considering the experimentally obtained C≈9.28 and ∆E/k≈1102 °K values for NaYF_4_:Yb^3+^/Er^3+^ materials with a hexagonal phase [40], the FIR yields characteristic exponential growth, as shown in Figure 1b.

Despite the exponential character of Equation (1), notice that, for temperature ranges around room temperature, 300 to 325 °K (25 to 50 °C), FIR can be approximated to a linear dependence with a slope of $m=\;[FIR\left(\left(50\;{}^{\circ}C\right)\right)-FIR\left(25\;{}^{\circ}C\right)]/∆T\approx 0.003$ with ∆T=25 °C. This approach has already been used for “single-point” UC-based temperature measurements [12,18], an ideal temperature range for biological applications [18]. Therefore, to evaluate the potential use of FIR measurements using LSFM in such biological applications, we will focus our experiments on this specific temperature range.

## 3. Materials and Methods

### 3.1. Samples

**Figure 2:**
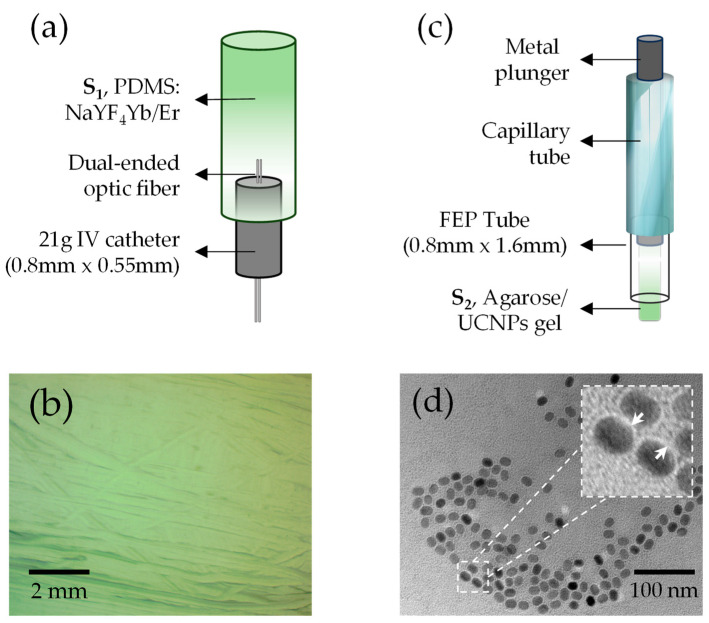
UC samples prepared for temperature mapping. (**a**) Large sample (*S*_1_) consisting of a cured mixture of PDMS: NaYF4:Yb^3+^/Er^3+^, a dual-ended optic fiber for signal excitation and detection, and a catheter for support. (**b**) A 4× reflection image of the S1 surface. (**c**) Short sample (*S*_2_) consisting of NaYF4:Yb^3+^/Er^3+^ nanoparticles immersed in agarose gel; the gel is suctioned inside the FEP tube with the help of a metal plunger, and a capillary tube is used for support. (**d**) TEM-micrograph of UCNPs@lipids dispersed in Mili-Q water. Inset: individual lipid-coated UC nanoparticles. White arrows indicate the lipid coating. Dashed square area: 50 × 50 nm^2^.

Two different kinds of samples, depicted in Figure 2, were prepared for temperature mapping, referred to as samples *S*_1_ and *S*_2_, for the large (microns to millimeters) and short (sub-microns to microns) range, respectively.

Figure 2a shows a diagram of the *S*_1_ sample consisting of a temperature sensor studied earlier by Sanchez-Escobar [12]. The sensor was made of a UC fluorescent composite containing standard polydimethylsiloxane, PDMS (Dow Corning, Midland, MI, USA, Sylgard 184), and a powder of sodium yttrium fluoride co-dopped with rare-earth elements (NaY_0_._77_Yb_0_._20_Er_0_._03_F_4_, Sigma Aldrich 756555-25G, St. Louis, MO, USA). The choice of PDMS as a host matrix for the NaYF4:Yb/Er particles was based on its broad-band optical transparency [42]. In particular, the optical absorption within the pump wavelength (980 nm) and the upconversion emission of the nanoparticles are low and thus avoid quenching the fluorescence bands. The fluoride powder was added to the PDMS in a concentration of 1% per weight to obtain the mixture. Since the powder contains particles around 1 to 5 μm in size, to avoid cluster formation due to its high hydrophobicity, 1 mL of chloroform (CHCl_3_) was added per gram of PDMS. The curing agent for the PDMS was added to the fluorescent composite in a 1:10 ratio, and both were mixed by hand for 3 min. The resulting solution was mixed with a magnetic stirrer heated to 60 °C until the CHCl_3_ evaporated. The mixture was subsequently poured into a polyvinyl chloride (PVC) tube (2 cm long, 2 mm external diameter, 1 mm internal diameter) used as a mold with a 21g catheter containing a dual-ended optic fiber placed inside, and the solution was heated at 75 °C for 1 h for solidification. As a final step, the PVC tube was removed upon immersion in acetone for 3 min, exposing the temperature-sensitive polymer compound. The UC material was evenly dispersed during fabrication in the PDMS without any visible clusters, indicating high-quality samples. This is shown in Figure 2b, acquired under 4× magnification.

Meanwhile, Figure 2c displays a diagram of the *S*_2_ sample consisting of agarose phantoms with lipid-coated UC NPs. It was prepared using commercial NaYF_4_:Yb^3+^/Er^3+^ UC nanoparticle (Sigma Aldrich, No. 900556 1 ML) colloid, containing 10 mg of 15 to 20 nm diameter NPs in 1 mL of Toluene. To facilitate their dispersion in an aqueous medium and to be suitable for biomedical applications, a lipid coating was applied to the UCNPs (UCNPs@lipids) because of their highly hydrophobic nature. We used a thin film hydration method involving three lipids, DOPS, cholesterol, and DMPC, at a molar ratio of 64:29:7, respectively. Based on Rojas-Gutierrez’s work [43], we chose a lipid ratio similar to that used in their study, which demonstrated the ability to form a lipid bilayer even in irregularly shaped UCNPs. The three lipids were dispersed in chloroform and mixed with 100 µL of UCNPs in a round-bottomed flask. The mixture was then evaporated under a constant flow of N_2_ while stirring in a circular motion for 30 min. Once the solvent was evaporated entirely, 2 mL of Milli-Q water was added to the flask and left to rehydrate overnight at 4 °C. The colloid solution of UCNPs@lipids was recovered at 1 mg/mL concentration and stored in microcentrifuge tubes (Eppendorf, Hamburg, Germany) at room temperature for future use and characterization. The TEM micrograph in Figure 2d confirms that the resulting lipid-coated nanoparticles were water-dispersible since no evident NP aggregates are observed. To create the end *S*_2_ agarose phantoms, 250 µL of UCNPs@lipids solution was mixed with 250 µL of 1× low-melting agarose gel. While the solution was still in a liquid phase, a few microliters of it were drawn up into an FEP tube (0.8 mm ID × 1.6 mm OD). Then, a part of the FEP tube was put inside a capillary tube to provide support, and the mixture was left to solidify for 30 min before imaging. Upon mixing the UCNPs@lipids colloid solution with agar, liposomes of approximately 16 µm size containing UCNPs were obtained, as shown later in the results section.

### 3.2. Light-Sheet Microscopy

**Figure 3:**
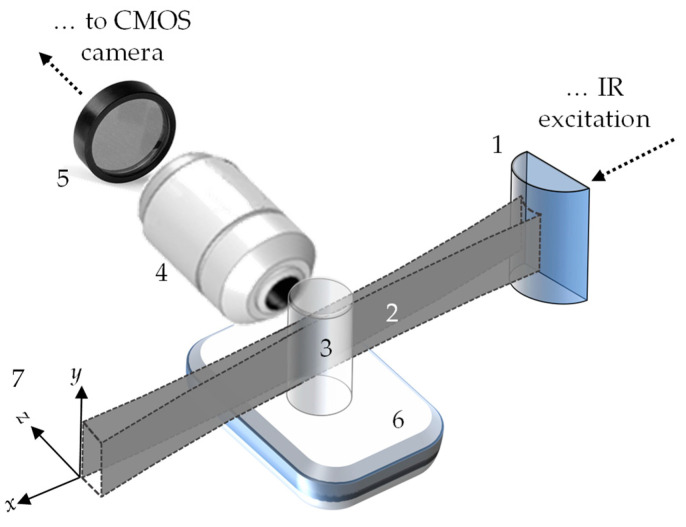
Generic light-sheet layout. The numbers in the figure stand for cylindrical lens (1), light-sheet (2), NaYF4:Yb^3+^/Er^3+^ sample (3), collection objective (4), optical filters set (5), temperature control system (6), and *xyz* motion control system (7) for sample movement. The *xy*-plane is defined as the image plane (plane of interest), and the *z*-axis is the direction orthogonal to that plane along the path where the fluorescence is collected.

Figure 3 shows a generic design for the light-sheet imaging system used for our experiments. By using cylindrical optics, it is possible to create planar illumination that can selectively excite a specific plane within the sample (which is why this light-sheet modality is also known as SPIM, standing for selective plane illumination microscopy). Then, a CMOS camera images the resulting planar fluorescence through an objective lens placed orthogonally to this plane. The excitation light is filtered out, and the fluorescence signal is transmitted using interferometric passband filters. This technique enables the fast imaging of the different samples’ fluorescence planes via simple depth scanning for further 3D reconstruction. In this work, two different SPIM systems were implemented to visualize the large *S*_1_ and the short *S*_2_ samples, which are described below.

The first SPIM system, used for imaging the *S*_1_ samples, utilized a continuous wave (CW) fiber-coupled laser diode (G&H, MA, USA, Single-Mode, AC1409) at 980 nm wavelength for efficient UC-fluorescence excitation, and the light-sheet was generated using an achromatic cylindrical lens (ACY254, Thorlabs, Newton, NJ, USA) of 50 mm in focal length. The measured light-sheet thickness along 1 mm of the cylindrical lens focal depth was around 57 µm. We used a temperature-controlled incubator system (Okolab, Pozzuoli, Italy, UNO-COMBINED), including a regulating unit and a heated chamber, to heat the sample. The chamber’s temperature can be precisely controlled within the 25 to 50 °C range with an accuracy of 0.3 °C. We attached the *S*_1_ to the chamber using thermal tape. We used a digital thermometer (Omega, Norwalk, CT, USA, RDXL4SD) equipped with two thermistors to monitor the temperature during the experiment. One thermistor was placed in the chamber, while the other was placed on the sample. Both temperatures were monitored until they matched in order to proceed with the fluorescence acquisition, and this procedure was repeated for every temperature set to heat the sample. The collection path used a high magnification optical system (Thorlabs, Newton, NJ, USA, MVL6X12Z, with an MVL20A extension tube) placed orthogonally to the sample, which projected the image onto a CMOS camera (Thorlabs, DCC3240C). This system has a magnification range from 1.4 to 9× with a respective numerical aperture (NA) range of 0.023 to 0.071. Given the large sample size, we used the lowest magnification; therefore, considering the well-known Rayleigh criterion for the optical resolution limit, δ = 0.61λ/NA, with a wavelength λ = 550 nm, the resulting lateral resolution of this SPIM system was approximately 15 µm. The camera sensor is 1280 × 1024 pixels^2^, covering an amplified image of 4.8 × 3.8 mm^2^ (0.27 pixel/µm). The temperature-controlled chamber was mounted on a linear translation stage (Newport, Irvine, CA, USA, M-UTM100PE.1) to capture depth images, which moved along the *z*-axis perpendicular to the sample’s *xy*-plane. To acquire the bidimensional FIR measurements, we used two interchangeable filters (Thorlabs, MF510-40 and MF559-34) to select the specific fluorescence emission bands at 525 and 550 nm. An extra passband filter (FES0750, Thorlabs) was also used to block the remaining excitation light.

The second SPIM system, employed for *S_2_* sample imaging, utilized a fiber Bragg grating stabilized pump module laser (JDSU, San Jose, CA, USA, 2900 Series) at 980 nm for excitation. To create the light-sheet, previously collimated IR light was focused along one dimension (the *z*-axis) onto the back focal plane of the excitation objective lens (Nikon, Tokyo, Japan, PlanFluor, 4×/0.13 NA, Nikon) by using a cylindrical lens with a focal length of 250 mm (Thorlabs, LJ1267RM-A). The excitation objective’s output forms the selective illumination plane at a sample’s *xy*-plane. A custom-made immersion chamber holds the capillary glass tube where the S_2_ agarose phantoms are immersed from the top. The capillary glass was attached to an *xyz*-motorized stage (3-axis NanoMax, Thorlabs), allowing for depth image acquisition. The UC image was captured using a highly sensitive digital CMOS camera (Hamamatsu, Shizuoka, Japan, ORCA-Flash4.0 LT3, C11440-42U40) combined with a water immersion objective lens (Nikon PlanFluor, 10×/0.3 NA) and a regular tube lens that provided the objective design magnification. This SPIM system produces a field of view (FOV) of approximately 1.3 × 1.3 mm^2^, with an optical axial resolution of roughly 1.3 µm. The same optical interferometric filters (Thorlabs, MF510-40, and MF559-34) were used to individually image the UC fluorescence emission bands at 525 and 550 nm. In contrast to the other setup, a bandpass filter (Thorlabs, FESH-0650) filtered out the excitation light instead. To heat the immersed sample, a custom-made temperature-controlled water recirculating system was used to heat the chamber. The system consisted of a peristaltic pump (KF Technology, Roma, Italy, NE-9000) and a solution heater (Single Inline Solution Heater, Warner Instruments, Hamden, CT, USA) with a temperature controller (Warner Instruments, TC-324C), allowing for temperature control from 25 to 50 °C with an accuracy of 0.2 °C.

## 4. Results and Discussions

### 4.1. FIR Characterization from UC Spectra

**Figure 4:**
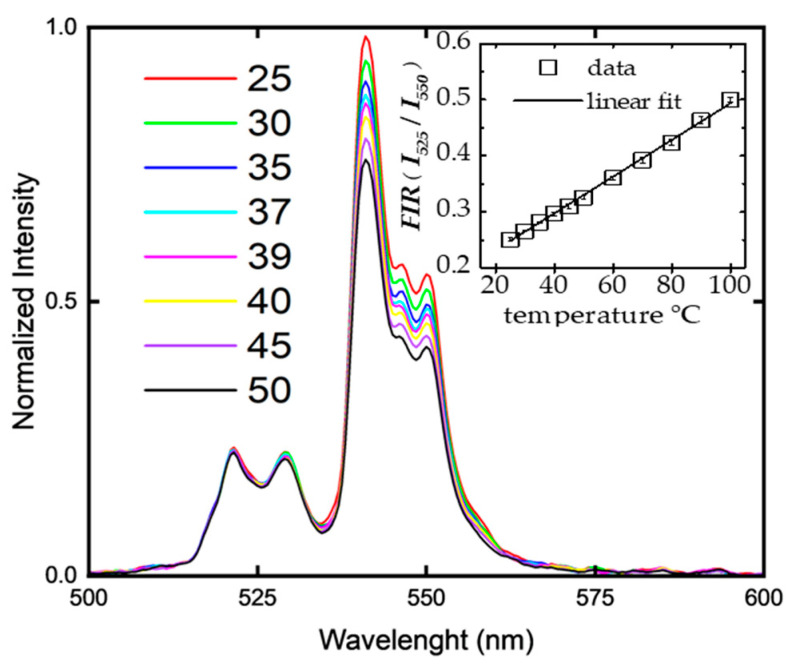
Conventional FIR characterization from temperature-dependent UC fluorescence spectra. Inset: FIR temperature dependence data (squares) and fit (solid line) resulting in the linear equation (Equation (2)) (see text) FIR = 0.0033T + 0.16426.

Sample *S*_1_ underwent *FIR* characterization before undergoing two-dimensional temperature experiments, following the standard methodology for a single-point *FIR* measurement outlined in Figure 4. UC spectra were taken while heating the sample to different temperatures covering a range from 25 to 50 °C. The laser source at 980 nm was used for UC fluorescence excitation at a constant power of approximately 100 mW, which was measured after focusing the light on the sample. The obtained spectra confirm that the integrated intensity of the higher energy band centered at 525 nm slightly increases, while the counterpart lower-energy-intensity band centered at 550 nm decreases. The temperature-dependent intensity ratios between the thermally coupled bands at 525 nm and 550 nm were then obtained and plotted as a function of temperature for a range of 25 to 100 °C (see figure inset), resulting in the linear equation

(2) FIR(T)=0.0033T+0.1646

This equation (Equation (2)) is consistent with previous results [12] and will be used here as a calibration curve for the two- and three-dimensional *FIR* experiments using light-sheet microscopy, shown next.

### 4.2. Two-Dimensional FIR Obtention Using Light-Sheet Imaging

**Figure 5:**
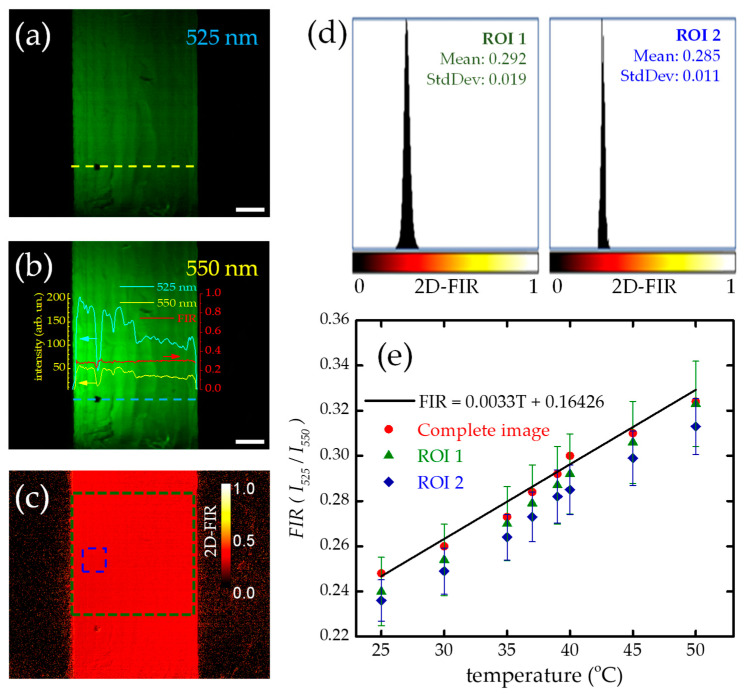
Bidimensional FIR characterization using light-sheet imaging. (**a**,**b**) UC fluorescence images of the 525 and 550 nm bands taken at 40 °C. Inset: Intensity profiles for the 525 (dashed yellow line) and 550 nm (dashed cyan line) images. The red curve is the FIR obtained when dividing the 525 nm (cyan) over the 550 nm (yellow) intensity profile. (**c**) The resulting 2D-FIR image shows two regions of interest: ROI 1 (dashed green square) and ROI 2 (dashed blue square). The red-hot scale from 0 to 1 indicates the 2D-FIR values. (**d**) Histograms for 2D-FIR values at ROI 1 (left) and ROI 2 (right). (**e**) A 2D-FIR temperature dependence from 25 to 50 °C for the complete image (red circles), ROI 1 (green triangles), and ROI 2 (blue rhombs) showing good agreement with the Equation (2) FIR calibration curve (black solid line). Supplementary Video S1: Two-dimensional FIR temperature dependence depicting (**a**–**e**) a temperature range of 25 to 50 °C.

Figure 5 summarizes the methodology proposed here to measure two-dimensional FIR using light-sheet microscopy. Figure 5a,b show the UC fluorescence images at the 525 and 550 nm bands of a single plane of sample *S*_1_ taken at a 40 °C temperature. As expected, the image at the 550 nm band is of higher intensity than that at the 525 nm band. This can be appreciated in the Figure 5b inset, which displays the UC fluorescence signal profiles for the yellow-dashed and cyan-dashed lines on the 525 and 550 nm images, respectively. The result of dividing these two signal profiles is shown in the red trace. Meanwhile, Figure 5c shows the two-dimensional FIR obtained by dividing the whole images (Figure 5a,b), where the 2D-FIR value is represented by a red-hot colormap ranging from zero to one, with zero being the minimum FIR value and one being the largest. It is important to note that if the FIR value of a pixel is close to one, there could be two possible scenarios. First, there may be no UC fluorescence emission in the region because of a lack of UC material. In this case, both the 525 and 550 intensities (*I*_525_ and *I*_550_) will be close to values of zero, but the intensity ratio will be close to one. Second, both bands may reach the same intensity value, but this can only happen at very high temperatures where UC signal quenching occurs (>200 °C) [44]. The first scenario is more likely to happen in our experiments since the maximum temperature we used for this experiment was 50 °C. Therefore, it is reasonable to conclude that the particles are not uniformly distributed within the sample. Such a lack of UC material will be confirmed later with FIR volumetric imaging.

Despite the apparent good UC material homogeneity obtained from the *S*_1_ sample (see Figure 2b), the 2D-FIR values show a slight variation depending on the size of the selected area. This can be observed in Figure 5c by inspecting the three regions of interest (ROIs) shown in the figure: the entire image (including the area outside the sample), a green dashed line box for a large area within the sample, and a blue dashed line box for a smaller size. Notice that the larger the area, the more dispersed the 2D-FIR values, as demonstrated in Figure 5d**,** which depicts 2D-FIR histograms for ROI 1 and ROI 2. While the FIR mean value for the former ROI is 0.292, for the latter, it is 0.285; both values are close to the FIR = 0.3 value obtained by the Equation (2) calibration curve for T = 40 °C.

The results presented in Figure 5e show that the temperature-dependent mean values of the 2D-FIR obtained by imaging the UC sample separately at the two thermally coupled bands using light-sheet microscopy are consistent with the results obtained with traditional characterization methods (as shown in Figure 4) over a temperature range of 25 °C to 50 °C. For the three regions of interest shown in Figure 5c, the data dispersion for the complete image (red circles), ROI 1 (green triangles), and ROI 2 (blue rhombs) lie within the characterization curve depicted in black solid lines.

### 4.3. Three-Dimensional Temperature Mapping

Since light-sheet microscopy can acquire 3D images by optically sectioning individual planes at different FOV and thickness scales, it is possible to create volumetric temperature maps from the samples prepared here at both millimeter and micrometer scales, as demonstrated below.

#### 4.3.1. Large Sample: *S*_1_

**Figure 6:**
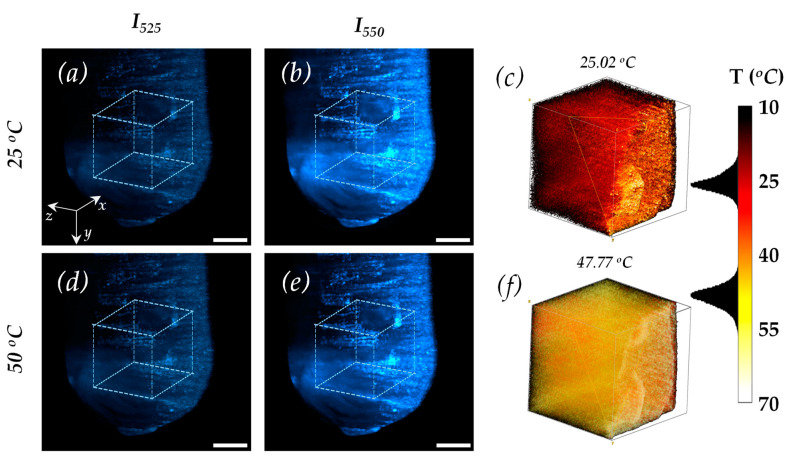
Three-dimensional reconstruction of the UC fluorescence signal and temperature of *S_1_*. (**a**,**b**) Volumetric UC- images for 525 and 550 nm bands taken at 25 °C. (**d**,**e**) Volumetric UC- images for 525 and 550 nm bands taken at 50 °C. The scale bar in (**a**,**b**,**d**,**e**) is 50 microns. The 3D temperature reconstructions for 25 °C (**c**) and 50 °C (**f**) agree with the sensed mean value of the temperature. The red-hot colormap scale in Figure (**c**,**f**) indicates the computed temperature using the calibration equation (Equation (2)).

Figure 6 shows the three-dimensional reconstruction of the UC fluorescence signal and temperature of *S*_1_. Figure 6a,b show the UC fluorescence image of the *I*_525_ and *I*_550_ bands at 25 °C. Meanwhile, Figure 6d,e display the images at 50 °C. Note that, as in the spectral case, the characteristic thermal coupling of the energy bands can be observed in these images when the temperature increases. To follow this phenomenon, let us focus on the 1 mm^3^ cube outlined with segmented lines in each figure. The UC fluorescence intensity exhibits a slight increase when the temperature is raised from 25 °C (Figure 6a) to 50 °C (Figure 6d). This increase is observed explicitly for the higher energetic band, *I_525_*. Meanwhile, the less energetic band at *I*_550_ displays a decrease in UC fluorescence intensity as the temperature increases from 25 °C (Figure 6b) to 50 °C (Figure 6e). Notice that the UC fluorescence images provide further insights into the homogeneity of the sample. They show the formation of structures of approximately 15 μm, indicating small clustering in the material. It is noteworthy that the utilized SPIM system has, in fact, a 15 μm resolution limit (as mentioned above) and, therefore, cannot resolve smaller structures. Since this accumulation of structures was observed only on one side of the sample, it could be attributed to the material precipitation from the micrometer-sized UC particles during the cooling process while preparing the mixture of PDMS and UC material. Furthermore, the decrease and even absence of a UC signal in some regions within the volume in the three-dimensional images shown in Figure 6a,b,d,e confirm both the inhomogeneity or lack of UC material within the sample.

Figure 6c,f demonstrate the close match observed between the sensed three-dimensional temperature and the applied temperature. We utilized Equation (2) as the calibration curve to determine the temperature at each voxel, and the hot-red scale shown in the figures indicates the calculated temperatures. The temperature registered within the volume matches the temperatures applied in order to heat the sample. When the applied temperature was 25 °C, a temperature distribution with a central value of 25.02 °C was detected (Figure 6c). Similarly, when a temperature of 50 °C was applied, the average temperature detected was 47.77 °C. However, this discrepancy could be due to the temperature not reaching equilibrium within the sample. Additionally, it should be noted that since the light-sheet scatters at the border of the sample, the UC fluorescent measurement in both bands is also affected. This leads to the acquisition of inaccurate temperature values since the voxels of each image do not necessarily align in space.

#### 4.3.2. Short Sample: *S*_2_

**Figure 7:**
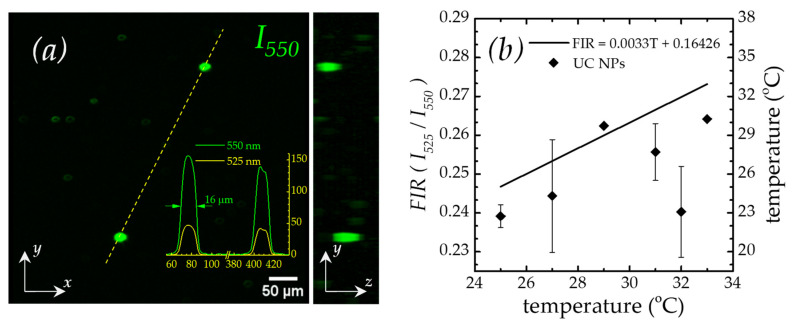
3D-FIR characterization of UC NPs using light-sheet imaging. (**a**) z-projection image of the UC fluorescence at 550 nm. Left: *xy*-plane. Right: *zy*-plane. Inset: UC fluorescence intensity plot profiles of the 525 nm (yellow) and 550 nm (green) bands. (**b**) 3D-FIR and temperature dependence from 25 to 33 °C.

Figure 7 shows the 3D-FIR characterization of *S_2_* in a temperature range of 25 to 50 °C. The UC fluorescence image in Figure 7a shows that the lipid-coated NPs agglomerated, forming round micrometric structures that, according to our assessment, appeared to be macro-liposomes. The left side of this figure shows the *z*-projection of the UC fluorescence intensity average across all *xy*-planes, while the right side shows the *z*-projection onto the *zy*-planes. The approximate image depth was around 100 μm. The figure inset shows the intensity profiles of the 525 nm (yellow line) and 550 nm (green line) intensity bands, from which one can see that the liposome’s formed diameter is approximately 16 μm. Meanwhile, Figure 7b demonstrates that the FIR obtained over the UCNPs@lipids shows a similar trend to that of the calibration curve of Equation (2); in fact, the data fit for the same slope. However, note that the FIR values and, hence, the temperature calculation are slightly lower. We could attribute this to the possibility that the actual temperature of the NPs did not reach thermal equilibrium with the externally applied temperature used to heat the sample. This situation may arise given that the NPs were immersed in agar, whose thermal properties are different compared with those of PDMS. In particular, the thermal conductivity of agar is reportedly higher (0.5 W/mK) than that of PDMS (0.2 W/mK) [45,46]; this will make the former host more prone to convective effects, thereby affecting the time required to reach thermal stability.

## 5. Conclusions

The acquisition of two-dimensional and three-dimensional temperature maps at different dimensional scales is possible by simply acquiring, through the light-sheet microscopy technique, upconversion fluorescence images of the two thermally coupled characteristic bands emitted by UC materials based on rare-earth ion (Er^3+^ and Yb^3+^) co-doping. This material and imaging technique combination allows for the measurement of volumetric temperature with high spatial and temporal resolution. Its use can be extended to biomedical applications, such as measuring internal temperatures in large biological systems (such as nematodes) for long ranges or small biological systems (such as cells) to monitor intracellular temperature. Therefore, we anticipate more experiments in this direction.

## Data Availability

The data presented in this study are available on request from the corresponding author.

## Supplementary Materials

The following supporting information can be downloaded at https://www.mdpi.com/article/10.3390/mi14112097/s1: Video S1: Two-dimensional FIR temperature dependence.
