# Supplementary figures and images for: Volumetric Temperature Mapping Using Light-Sheet Microscopy and Upconversion Fluorescence from Micro- and Nano-Rare Earth Composites
Source: Micromachines (Basel). 2023 Nov 14;14(11):2097. doi: 10.3390/mi14112097 (PMC10673603; doi:10.3390/mi14112097)

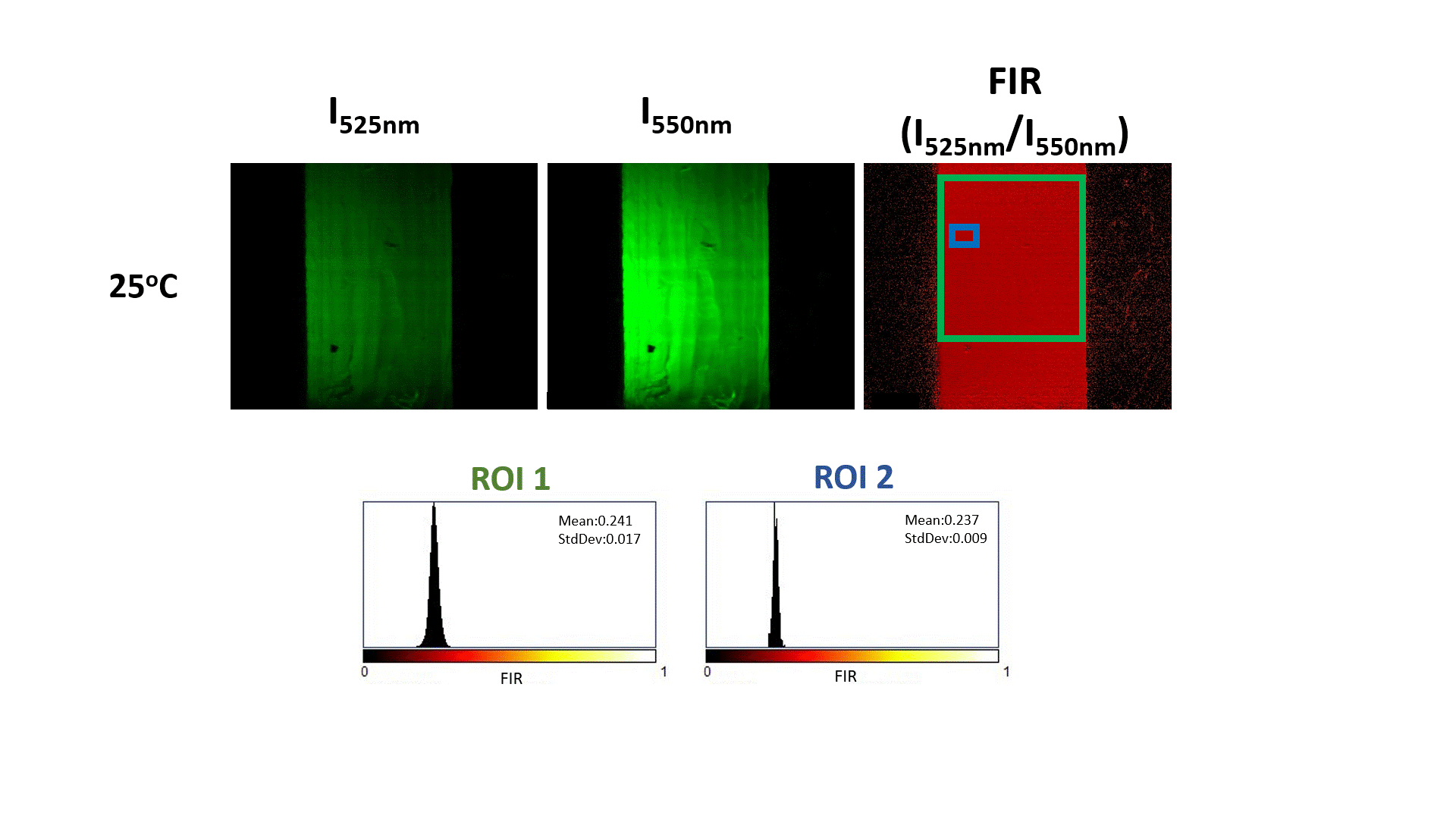

Supplement: Supplementary file 1 [file micromachines-14-02097-s001.zip › micromachines-2696042-supplementary.gif]
